# A Rare Case of Sarcomatoid-Type Localized Pleural Mesothelioma Derived From Visceral Pleura

**DOI:** 10.7759/cureus.81473

**Published:** 2025-03-30

**Authors:** Takayuki Kosaka, Seshiru Nakazawa, Rikuo Ushikubo, Takashi Ibe, Ken Shirabe

**Affiliations:** 1 Department of Thoracic Surgery, National Hospital Organization (NHO) Takasaki General Medical Center, Gunma, JPN; 2 Department of General Surgical Science, Gunma University Graduate School of Medicine, Gunma, JPN

**Keywords:** localized pleural mesothelioma, lung cancer, mesothelioma, pleural mesothelioma, sarcomatoid-type pleural mesothelioma

## Abstract

A man in his 80s presented with shortness of breath. Chest CT scan showed pleural effusion and a mass in the right lower lobe. He had high inflammatory reaction and was diagnosed as having pulmonary suppuration with empyema. After three weeks of antibiotic treatment, the lesion grew and he was referred to our department for surgical intervention. A complete resection of the right lower lobe was planned to control infection. Intraoperative findings showed that the lower lobe became a large mass and was judged unresectable. The procedure was converted to drainage of the abscess cavity. Pathological examination, combined with clinical and radiological findings, led to the diagnosis of localized sarcoma-type malignant pleural mesothelioma. Localized malignant pleural mesothelioma is a rare disease and most cases have been reported as epithelial type, deriving from the parietal pleura. Our case is quite rare because it was a sarcoma-type, deriving from the visceral pleura.

## Introduction

Localized pleural mesothelioma (LPM) is a rare disease that does not show diffuse development [1,2]. Most cases have been reported as epithelialoid-type, deriving from the parietal pleura. Here, we report a case of sarcomatoid-type LPM deriving from the visceral pleura that was preoperatively misdiagnosed as pulmonary suppuration because of high inflammatory reaction and pleural effusion.

## Case presentation

**Figure 1:**
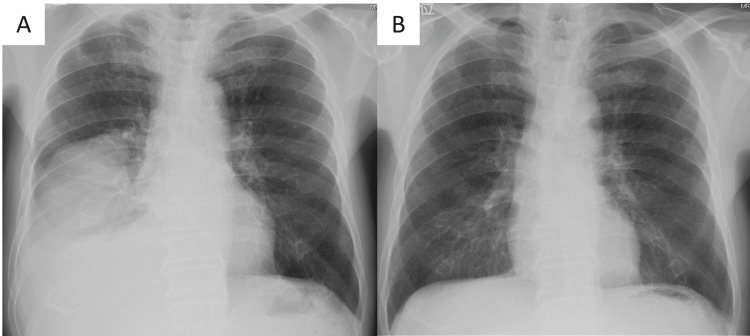
Chest X-ray findings of chest of first admission and one year ago Chest X-ray showed huge mass in the right lower lung field (A), which had not been detected one year ago (B).

**Figure 2:**
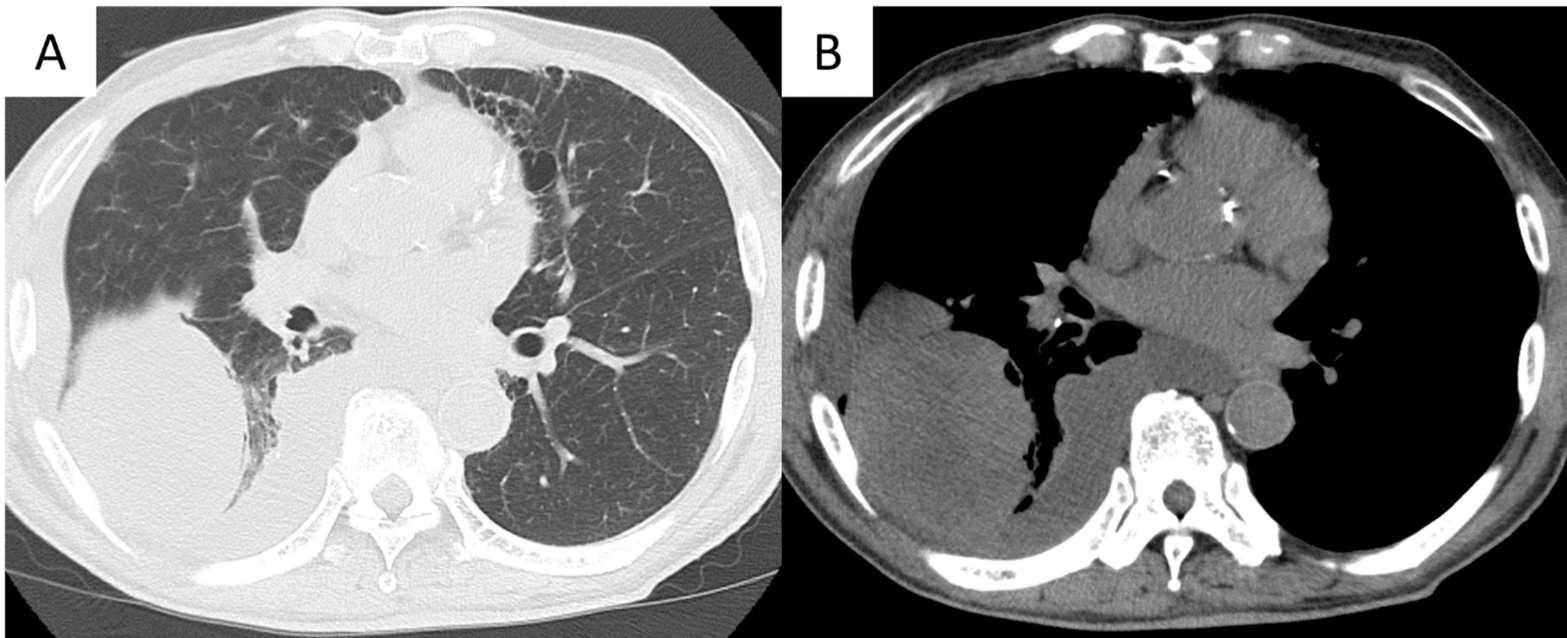
Chest computed tomography findings of chest at first admission Non-contrast chest computed tomography scan at first admission showed moderate amount of pleural effusion and a mass of 60x50x80 mm with heterogeneous internal density in the right lower lobe. A: pulmonary window, B: mediastinal window

A man in his 80s visited a local hospital with shortness of breath that had continued for a week as his chief complaint. He had comorbidities of hypertension, diabetes, and chronic obstructive pulmonary disease. He was a current smoker with a smoking status of 90 pack-year. He had never been exposed to asbestos. Chest X-ray showed a huge mass in the right lower lung field (Figure 1A), which had not been shown one year ago (Figure 1B). Non-contrast chest computed tomography (CT) scan showed moderate amount of pleural effusion and a mass of 60x50x80 mm with heterogeneous internal density in the right lower lobe (Figure 2A, 2B). He also showed moderate inflammatory reaction (WBC count: 9130/µL (reference range: 3300-8000), C-reactive protein (CRP) concentration: 2.5 mg/dL (reference range: <0.14)). He was admitted to the hospital and underwent intercostal tube drainage. The pleural effusion was yellow and cloudy, and diagnosed as class Ⅱ by cytology. He was diagnosed as having pulmonary suppuration with empyema and underwent antibiotic treatment. Although the treatment was continued for two weeks, the inflammatory reaction got worse (WBC count: 11160/µL, CRP concentration: 13.0 mg/dL), and CT images showed that the mass increased in size. Because there was no improvement after three weeks of antibiotic treatment, he was referred to our department for surgical intervention.

**Figure 3:**
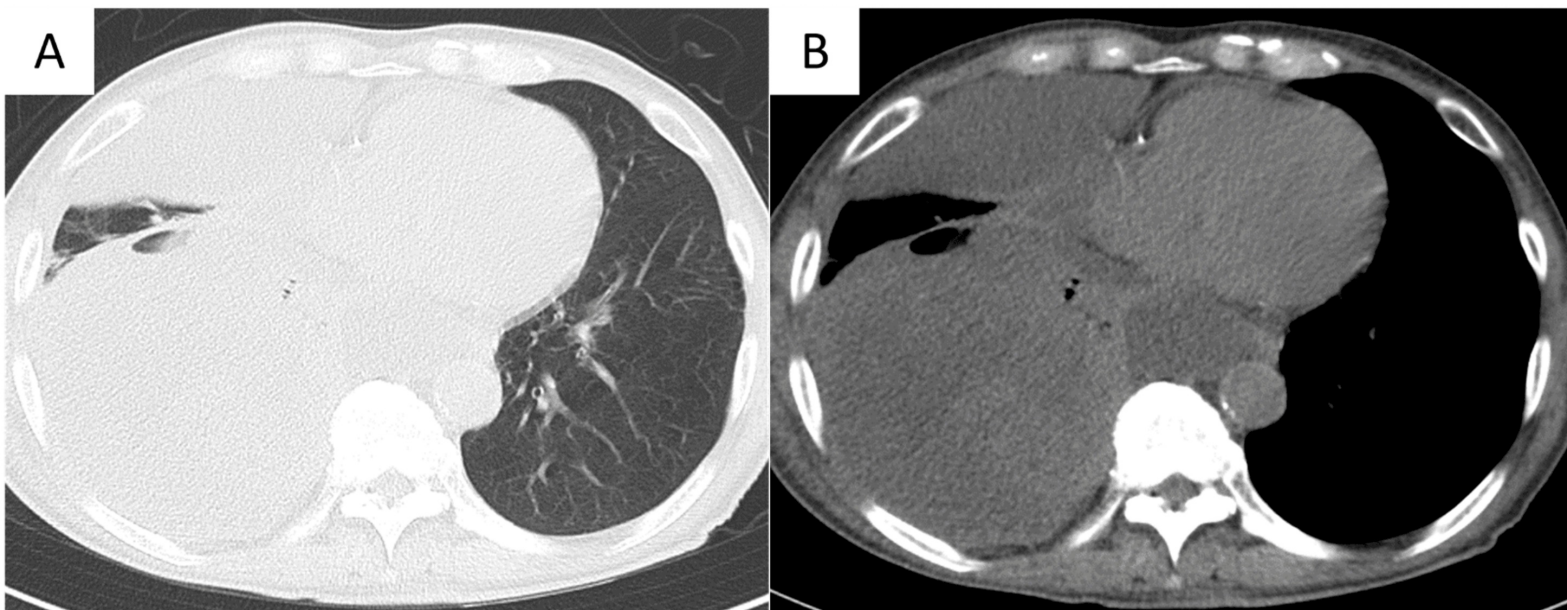
Chest computed tomography findings of chest at admission to our hospital Non-contrast chest computed tomography scan at admission to our hospital showed the right lower lobe lesion had increased in size, up to 110x110x150 mm, with fluids within the mass. A: pulmonary window, B: mediastinal window

Blood examination in our hospital showed high inflammatory reaction (WBC count: 18500/µL, CRP concentration: 25.41 mg/dL). There was no elevation of tumor markers (carcinoembryonic antigen, cytokeratin 19 fragment, and pro-gastrin-releasing peptide) or markers of infectious disease (tuberculosis, aspergillus, and fungus). Chest CT showed the right lower lobe lesion had increased in size, up to 110x110x150 mm, with fluids within the mass after four weeks from the first CT (Figure 3A, 3B). Our diagnosis was pulmonary suppuration or lung cancer with infection, and surgical intervention was planned to control infection. Any imaging studies to search for distant metastases other than chest CT were not performed because removal of the cause of the inflammation as soon as possible, even if it was caused by the tumor. Complete resection of the right lower lobe was planned.

Intraoperative findings showed that the lower lobe became a large mass that occupied the lower part of the thoracic cavity, and was firmly adhered to the surrounding chest wall. Adhesion between the mass and the chest wall was separated. There seemed to be no invasion of the mass into the chest wall. However, because adhesion between the interlobar spaces was also strong, it was judged difficult to remove the lower lobe. Because right middle and lower lobectomy or pneumonectomy was considered difficult due to the high age and poor performance status of this patient, the procedure was converted to drainage of the abscess cavity. A thin part of the abscess wall was incised to open the abscess cavity as much as possible. A part of the abscess wall was also collected for postoperative pathological examination. After thorough washing of the thoracic cavity, a drain was placed and the surgery was terminated.

**Figure 4:**
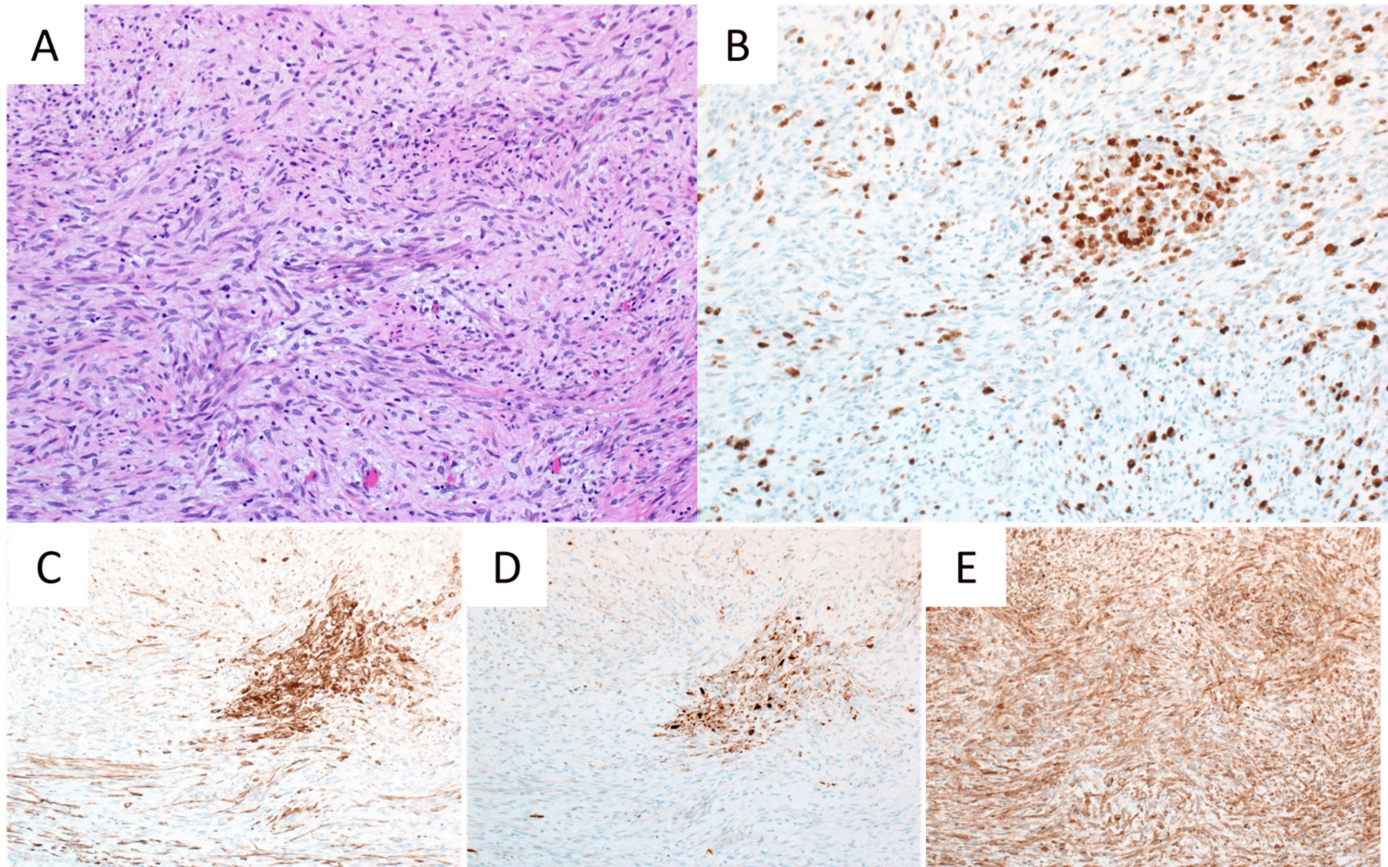
Microscopic findings of the tumor at 10x magnification (A) H&E stains showed spindle-shaped tumor cells with short oval nuclei containing small granular chromatin grew with fasciculation. (B) 70% of tumor cells were positive for Ki67. Immunohistochemical staining showed positive staining for keratin AE1/AE3 (C), keratin CAM5.2 (D), and CD99 (E).

Pathological examination showed spindle-shaped tumor cells with short oval nuclei containing small granular chromatin which grew with fasciculation (Figure 4A). Nuclear-cytoplasmic ratio was high and there were light anisonuclei. Many cells were at mitosis. The proportion of tumor cells positive for Ki67 was 70% (Figure 4B). Immunohistochemical staining showed positive staining for keratin AE1/AE3, keratin CAM5.2, and CD99 (Figure 4C, 4D, 4E, respectively). The tumor was diagnosed as sarcomatoid-type pleural mesothelioma. In addition, the tumor was judged to be of visceral pleural origin because intraoperative findings clearly showed there was no invasion to the parietal pleura. Furthermore, since no lesions other than the tumor were observed on thoracic and upper abdominal CT, the tumor was judged to be solitary. Based on the above findings, the tumor was diagnosed as a sarcomatoid-type LPM that was derived from visceral pleura.

Although the blood tests showed decrease in inflammatory markers and the pleural effusion disappeared postoperatively, the thoracic mass continued to increase in size. Chemotherapy was considered, but was not performed due to worsening performance status. The patient died two months after the operation.

## Discussion

We have herein reported a case of sarcomatoid-type LPM that was preoperatively diagnosed as pulmonary suppuration. The tumor growth was rapid and the patient died soon after diagnosis.

Localized mesothelioma (LM) is a rare disease and is defined as a solitary, nodular lesion, without diffuse involvement of the serosal surface, both macroscopically and histologically [1,2]. It is distinguished from diffuse mesothelioma (DM) because of its localized presentation, quite different biologic behavior, and better prognosis [3]. LM has been identified in only 0.5-1.6% of cases diagnosed as mesothelioma [4]. Until 2020, only 101 LM have been reported in the literature [4]. Most of the cases (82%) were described as localized thoracic tumors that presented as a pleural, chest wall, mediastinal, and/or pulmonary mass suspicious for a carcinoma or a mesenchymal tumor [4].

Mesotheliomas are pathologically divided into three subtypes: epithelioid, sarcomatoid, and biphasic. A review of 52 cases of LPM showed the frequency as epithelioid, 52.8%; sarcomatoid, 15.1%; biphasic, 32.1% [5]. The pleura of origin has been described in 13 reported cases; only two (15%) from the visceral pleura and 11 (85%) from the parietal pleura [5]. Patients' age ranged from 37 to 80 years; 79% were men, and tumors varied in size from 1.7 to 19 cm [5]. Our case is rare because it was a sarcomatoid-type, deriving from the visceral pleura. Asbestos exposure is considered one of the important risk factors of pleural DM (DPM). Compared to DPM, a history of asbestos exposure seems to be less frequent in LPM. Only 38.5% of cases had a history of asbestos exposure in the 52 reported cases of LPM [5]. Our case also had no history of asbestos exposure. The factors that cause the LPM may be different from DPM.

The prognosis of patients with LM has been reported to be considerably better than for patients with DM [4]. In addition, patients with epithelioid lesions had a significantly better survival than those with sarcomatoid or biphasic tumors [4]. However, more strict statistical analysis with larger cohort is needed to draw a real conclusion, as the authors described [4]. Several reports showed the possibility of curability by complete resection of LM [3,4]. The prognosis of our case was poor probably because it was a sarcomatoid-type LM and also due to its large size, which did not allow complete resection. If the tumor had been resected while it was still small, a better prognosis might have been achieved. Also, a high inflammatory reaction and pleural effusion caused by intratumoral infection made it difficult to distinguish from pulmonary suppuration before operation.

## Conclusions

We reported here a rare case of sarcomatoid-type LPM that was derived from visceral pleura. High inflammatory reaction and pleural effusion caused by intratumoral infection made it difficult to distinguish from pulmonary suppuration before operation. We should consider sarcomatoid-type LPM as one of the differential diagnoses when we see a rapidly growing mass in the thoracic cavity.
